# WHO Digital Health Guidelines: a milestone for global health

**DOI:** 10.1038/s41746-020-00330-2

**Published:** 2020-09-18

**Authors:** Alain Labrique, Smisha Agarwal, Tigest Tamrat, Garrett Mehl

**Affiliations:** 1 Department of International Health, Johns Hopkins Bloomberg School of Public Health, Baltimore, MD USA; 2 Department of Sexual and Reproductive Health and Research, includes HRP - the UNDP/UNFPA/UNICEF/WHO/World Bank Special Programme of Research in Human Reproduction, World Health Organization, Geneva, Switzerland

**Keywords:** Health services, Public health, Health policy

## Abstract

In 2019, the World Health Organization (WHO) released the first-ever evidence-based guidelines for digital health. The guideline provides nine recommendations on select digital health interventions that involve the use of a mobile phone or device. It also provides information on implementation considerations, quality and certainty of extant evidence, factors related to acceptability and feasibility of the intervention, and gaps in the evidence that can inform future research. Given the pivotal role digital health can play in supporting health systems, seen especially in light of the COVID-19 pandemic, these guidelines can help provide a roadmap for governments and policymakers in introducing and scaling up digital health interventions to support population health outcomes.

## Introduction

On April 17, 2019, the World Health Organization (WHO) reached a new milestone as it released the first evidence-based guidelines on digital health^1^. These nine recommendations were the culmination of a multi-year process, identifying, distilling, and synthesizing the evidence around the impact of digital interventions for health systems strengthening. The earliest period of mobile-phone augmented digital health, sometimes referred to as mHealth, was characterized by widespread use of phones to overcome persistent infrastructural and health service delivery challenges^2,3^. These new guidelines focus on Digital Health interventions that leverage a mobile phone or device^4^. This is a subset of the definition of Digital Health used in the May 2018 World Health Assembly Resolution which included a wide range of technologies across the spectrum of eHealth, mHealth, telemedicine, and even emerging areas of advanced computing such as “big data”, machine learning, and artificial intelligence. The WHO has played a critical leadership role in building consensus across the field of digital health, including characterizing the ways in which digital technologies are being used to support health needs^5^, proposing standard procedures unique to evaluating digital health interventions^6^, the establishment of a formal Department of Digital Health and now with these guidelines, proposing best practices for country governments to consider as they develop and scale digitized health systems^1^. While donor agencies and governments have both expressed keen interest in identifying ways to leapfrog gaps in infrastructure and leverage digital tools to enhance the coverage and quality of service delivery, much of the implementation has occurred in the absence of careful examination of evidence. The paucity of evidence in digital health requires the global digital health community to take a more deliberate and coordinated approach to identifying and addressing research gaps, perhaps as part of a global action plan guided by the needs of different stakeholders, principally Ministries of Health.

## Role of digital health in health systems strengthening

The Digital Health Guidelines followed the WHO evidence-to-decision framework^7^ systematically leveraging extant evidence as well as expert opinions across nine priority emergent digital innovations targeting at health systems strengthening^1^. The recommendations are summarized in Table 1. In addition to the list of recommendations on select interventions, the guidelines also highlight the quality and certainty of extant evidence, a series of implementation considerations for each of the highlighted approaches as well as factors related to the acceptability and feasibility of the interventions, and the gaps in evidence to inform future research directions^1^.

**Table 1:** Summary of WHO Guideline: recommendations on digital interventions for health system strengthening.

	Intervention^a^	Health conditions	Recommendation	Effect^b^	Certainty of effect range^c^
1	Birth notification via mobile devices	N/A	RECOMMENDED ONLY IN SPECIFIC CONTEXTS OR CONDITIONS^a^:	No effect	Very low
			In settings where the notifications provide individual-level data to the health system and/or a civil registration and vital statistics (CRVS) system, and the health system and/or CRVS system has the capacity to respond to the notifications.		
2	Death notification via mobile devices	N/A	RECOMMENDED ONLY IN THE CONTEXT OF RIGOROUS RESEARCH^a^:	No studies identified	Very low
			In settings where: the notifications provide individual-level data to the health system and/or CRVS system, and the health system and/or CRVS system has the capacity to respond to the notifications.		
3	Stock notification and commodity management via mobile devices	All	RECOMMENDED ONLY IN SPECIFIC CONTEXTS OR CONDITIONS^a^:	No effect	Very low
			Where supply chain management systems have the capacity to respond in a timely and appropriate manner to the stock notifications.		
4	Client-to-provider telemedicine	All	RECOMMENDED ONLY IN SPECIFIC CONTEXTS OR CONDITIONS^a^:	No effect, + effect	Very low to moderate
			To complement, rather than replace, the delivery of health services and in settings where patient safety, privacy, traceability, accountability, and security can be monitored.		
5	Provider-to-provider telemedicine	All	RECOMMENDED ONLY IN SPECIFIC CONTEXTS OR CONDITIONS^a^:	No effect, + effect	Very low to low
			Where: patient safety, privacy, traceability, accountability, and security can be monitored.		
6	Targeted client communication (TCC) across five population groups	Sexual, reproductive, newborn, child, and adolescent health	RECOMMENDED ONLY IN SPECIFIC CONTEXTS OR CONDITIONS^a^:	*Adolescents*: No effect, + effect; *Adult users*: No effect, + effect, − effect^d^; *Pregnant/post-partum women*: No effect, + effect; *Pregnant/post-partum women with HIV*: No effect, + effect; *Parents of children <5 years of age*: No effect, + effect	*Adolescents*: Very low to low; *Adult users*: Very low to low; *Pregnant/post-partum women*: Very low to moderate; *Pregnant/post-partum women with HIV*: Very low to moderate^e^; *Parents of children <5 years of age*: Very low to moderate
			Under the condition that potential concerns about sensitive content and data privacy can be addressed.		
7	Health worker decision support via mobile devices (CDSS)	All	RECOMMENDED ONLY IN SPECIFIC CONTEXTS OR CONDITIONS^a^:	No effect, + effect	Very low to moderate
			For tasks that are already defined within the scope of practice for the health worker.		
8	Digital tracking of patients’/clients’ health status and services via mobile devices	All	RECOMMENDED ONLY IN SPECIFIC CONTEXTS OR CONDITIONS^a^:	*Tracking+TCC*: No identified data; *Tracking+CDSS*: No effect, + effect; *Tracking+CDSS+TCC*: No effect	*Tracking+TCC*: N/A; *Tracking+CDSS*: Very low to moderate; *Tracking+CDSS+TCC*: Very low
			Where the health system can support the implementation of these intervention components in an integrated manner; For tasks that are already defined as within the scope of practice for the health worker; and Where potential concerns about data privacy and transmitting sensitive content to clients can be addressed.		
9	Provision of training to health workers via mobile devices (mLearning)	All	RECOMMENDED^a^:	No effect, + effect	Very low to low
			To complement, rather than replace, traditional methods of delivering continued health education and post-certification training.		

^*a*^*Recommended*: the intervention/option should be implemented; *recommended only in specific contexts or conditions*: the intervention is applicable only to the condition, setting or population specified in the recommendation, and should only be implemented in these contexts; *recommended only in the context of rigorous research*: Given the uncertainties in the intervention/option, implementation should be undertaken in the form of research to address unanswered questions.

^b^Pertains to positive, no effect, or harmful effect on a range of outcomes assessed, including:

*Client Interventions (4,6)*: unintended consequences, resource use, satisfaction/acceptability, utilization of health services, health behavior, status, and well-being.

*Health worker interventions (5,7,8,9)*: unintended consequences, resource use, satisfaction/acceptability, health worker performance, health worker skills/attitudes, health worker knowledge, clients’ utilization of health services, clients health behavior, status, and well-being.

*Health systems interventions (1,2,3)*: unintended consequences, resource use, satisfaction/acceptability, coverage of birth/death registration (1,2), timeliness of birth/death notifications (1,2), coverage of newborn or child health services (1,2), timeliness of newborn or child health services (1,2), availability of commodities (3), quality and timeliness of stock management (3).

^c^Certainty of effect pertains to very low, low, moderate, and high certainty evidence based on grading of the evidence using Grading of Recommendations, Assessment, Development, and Evaluation (GRADE)^7^.

^d^For a study on TCC among adult users conducted in a community setting in Bangladesh, it was determined that the digital intervention may increase the number of women who experience physical violence^12^.

^e^The only evidence graded to be of moderate quality suggested little to no difference of the intervention on the number of pregnant women adhering to prenatal anti-retroviral medication.

### Strengths, limitations, and next steps

Table 1 presents the recommendations for each of the interventions along with a summary note of the underlying evidence (positive effect, no effect, harmful effect), and the strength of the evidence classified using GRADE^8^. Qualitative evidence was also extensively reviewed and contributed to the formulation of the recommendations, lending specificity to the contextual considerations and caveats associated with each recommendation. Despite the rigorous process of evidence review undertaken to gather and assess the strength of the evidence, we see that several recommendations are made despite the presence of minimal evidence—even that being of very low to low certainty. This usually reflected a post-deliberation consensus position of the members of the Guidelines Development Group, that despite equipoise in the state of evidence around some of the domains reviewed, the level of risk associated with the intervention was low enough to recommend its discretionary use in appropriate settings, with adequate monitoring. The potential for improvements in process efficiencies, even in the absence of changes in health outcomes, were often considered—although evidence of financial or time savings were also often lacking.

The process of developing the guidelines brought important limitations to the forefront, notably a continued dearth of robust evidence, despite efforts to strengthen the quality and completeness of digital health reporting^9^. The Cochrane process can be seen by some as unforgiving, requiring that reported research meet stringent GRADE^8^ and CERQual criteria^10^ before it is considered in guideline development. We expect that the research recommendations generated during the guideline-development process will provide valuable insights on strengthening digital health evidence and serve as an impetus to drive up standards of evaluation and reporting in this emergent field. Given the inevitability of digitized health systems, it is important that future revisions of and expansion of these guidelines are able to provide evidence-informed pathways to implementation and scale of digital health interventions.

### Relevance for policymakers, donors, and implementers

The guidelines address not only the effectiveness for each of the interventions, but also highlight key considerations for acceptability, feasibility in varying contexts, considerations for gender, equity and rights, and use of resources associated with the intervention. Due to the limited evidence on resource use, it is difficult to assess the comparative effectiveness of digital interventions compared to possibly less expensive non-digital interventions. However, donors and policymakers may consider that investments in digital data collection and reporting systems will likely reduce inefficiencies in data transfer and entry inherent to paper systems. The guidelines provide a roadmap so that investments in such digital systems are not haphazard, but are driven by a systematic process that considers experience, evidence, and potential risks so that prior decades’ experience with non-sustainable investments in innovations is not repeated.

## Conclusion

These guidelines are the first step to guiding governments in scaling up digital interventions. Beyond the obvious importance of evidence-based guidelines to assist digital health investors and implementers, the commissioning of this work together with the establishment of a formal Department of Digital Health at WHO bring the field into a new era—where we are no longer toying around with “shiny, new objects”, but leveraging a serious, disruptive tool to improve healthcare and protect health. During the ongoing COVID-19 global pandemic these guidelines have proven valuable in guiding governments to identify effective strategies across the response continuum, from communicating information to populations and at-risk or infected individuals, to assisting first responders with symptom triage on the frontlines^11^. These “living” guidelines^1^ will continue to be shaped by new research and be responsive to newer technologies as they emerge. As with many innovations in the fields of public health and medicine, we fully expect early adopters to test and even scale the digital health solutions the future holds before the next guidelines are complete; still, mainstream adoption and more importantly, the complete transformation of digital innovations into standard practice will benefit from these and future guidelines.

## Data Availability

Data sharing not applicable to this article as no datasets were generated or analyzed during the current study.
